# LncRNA HCP5 : A Potential Biomarker for Diagnosing Gastric Cancer

**DOI:** 10.3389/fonc.2021.684531

**Published:** 2021-06-18

**Authors:** Shiyi Qin, Lei Yang, Shan Kong, Yanhua Xu, Bo Liang, Shaoqing Ju

**Affiliations:** ^1^ Department of Laboratory Medicine, Affiliated Hospital of Nantong University, Nantong, China; ^2^ Research Center of Clinical Medicine, Affiliated Hospital of Nantong University, Nantong, China; ^3^ Medical School of Nantong University, Nantong University, Nantong, China; ^4^ Department of Medical Ultrasonics, Affiliated Hospital of Nantong University, Nantong, China

**Keywords:** gastric cancer, GC, HCP5, biomarker, diagnosis, gastritis

## Abstract

**Background:**

It has been reported that long non-coding RNAs (lncRNAs) can be regarded as a biomarker and had particular clinical significance for early screening and gastric cancer (GC) diagnosis. Therefore, this study aimed to investigate whether serum HCP5 could be a new diagnostic biomarker.

**Methods:**

Filtered out the HCP5 from the GEO database. The specificity of HCP5 was verified by real-time fluorescence quantitative PCR (qRT-PCR), and then the stability of HCP5 was verified by room temperature storage and repeated freeze-thaw experiments. Meanwhile, the accuracy of HCP5 was verified by agarose gel electrophoresis (AGE) and Sanger sequencing. Simultaneously, the expression level of serum HCP5 was detected by qRT-PCR in 98 patients with primary gastric cancer, 21 gastritis patients, 82 healthy donors, and multiple cancer types. Then, the methodology analysis was carried on. Moreover, receiver operating characteristic (ROC) was used to evaluate its diagnostic efficiency.

**Results:**

qRT-PCR method had good repeatability and stability in detecting HCP5. The expression level of HCP5 in the serum of gastric cancer patients was remarkably higher than that of healthy controls, and it could distinguish gastritis patients from healthy donors. Besides, the expression of HCP5 was increased dramatically in MKN-45 and MGC-803. The FISH assay showed that HCP5 was mainly distributed in the cytoplasm of MKN-45 and BGC-823 cells. When HCP5 was combined with existing tumor markers, the diagnostic efficiency of HCP5 was the best, and the combined diagnosis of carcinoembryonic antigen (CEA), carbohydrate antigen199 (CA199), and HCP5 can significantly improve the diagnostic sensitivity. Besides, compared with the expression levels of thyroid cancer (THCA), colorectal cancer (CRC), and breast cancer (BRCA), serum HCP5 in gastric cancer was the most specific. Moreover, the high expression of serum HCP5 was related to differentiation, lymph node metastasis, and nerve invasion. The term of serum HCP5 after the operation was significantly lower than that of patients with primary gastric cancer.

**Conclusion:**

Serum HCP5 can be used as a potential biomarker of non-invasive fluid biopsy, which had a unique value in the early diagnosis, development, and prognosis of gastric cancer.

## Introduction

Gastric cancer (GC) is one of the most common malignant tumors globally, with high mortality and morbidity (1). As shown by data, 50% of gastrointestinal cancer cases have occurred in China, of which gastric cancer accounts for the most significant proportion, and the 5-year overall survival rate is meager, which was reported <35% in 2013-2015 (2). Among several factors, helicobacter pylori infection was the most common one, including other high-risk factors like genetic susceptibility, diet, drinking, smoking, etc. (3). At this stage, gastric cancer treatment mainly relies on surgical techniques, traditional radiotherapy, chemotherapy, and neoadjuvant therapies. However, since GC is rarely diagnosed early, it has usually advanced to most patients’ late stages. Furthermore, traditional surgery, radiotherapy, and chemotherapy are often not as effective as early-stage disease, malignant invasion, and metastasis later appeared (4). That means it is urgent to find a screening index with high specificity and sensitivity. Studies have shown that molecular analysis of non-invasive body fluids may help diagnose GC (5). In recent years, more and more literature has demonstrated that long non-coding RNA (lncRNA) played an essential role in the pathogenesis and process of GC (6–8). Hence, lncRNA has become the focus of attention in recent years.

LncRNA is a non-coding RNA with a length of more than 200nt, lacking the potential of coding peptides (9). Increasing evidence has shown that lncRNA can regulate genes by directly binding to genes, participating in translation inhibition, splicing modification, and messenger RNA (mRNA) degradation, and using microRNA (miRNA) as a competitive endogenous RNA (ceRNA) to prevent mRNA degradation and stabilize mRNA (10, 11). It plays a vital role in the regulation of splicing, transcription control, and post-transcriptional processing (12). Moreover, lncRNA may act as tumor suppressor genes and oncogenes, affecting cell proliferation (13), apoptosis (14), differentiation (15), metastasis (16), DNA damage (17), angiogenesis (18), and immune response (19), and so on. Simultaneously, the existing literature has confirmed that lncRNAs also exist in serum, plasma, urine, and exosomes of various cancer types, indicating that they can also be used as classic markers for liquid biopsy. For example, FAM83H-AS1 and lncRNA-ATB were significantly high in serum breast cancer patients, showing the potential ability to monitor breast cancer progression and staging (20). Besides, lncRNA GAS5 resulted in a specific reduction of plasma expression in coronary artery disease (CAD) patients, which could be used as a specific biomarker for diagnosing CAD (21). What’s more, the up-regulation of LncRNA XIST transported by serum extracellular vesicles was related to the progression of colorectal cancer (CRC) (22). It was also reported HYMA1, OTX2-AS1 linc00477, and loc100506688 in urine exosomes, which were found in bladder cancer, could potentially serve as biomarkers and therapeutic targets (23). Consequently, lncRNAs could be used as molecular markers for tumor diagnosis and new targets for tumor treatment shortly (24).

Histocompatibility leukocyte antigen complex P5 (HCP5) is a momentous lncRNA located between MHC class I polypeptide related sequence A (MICA) and MHC class I polypeptide related sequence B (MICB) genes in the MHC class I chain-related gene (MHCI) region, involving many autoimmune diseases and malignant tumors (25). Moreover, the abnormal expression of HCP5 is closely related to cell proliferation, migration, invasion, apoptosis, lymphatic metastasis, and drug resistance in various cancers (26–29). Hence, HCP5 is expected to become a biomarker and therapeutic target in multiple cancers (25). Functional tests verified that HCP5 could function as an oncogene in osteosarcoma and was activated by the transcription factor 1 (SP1), convincing that SP1 induced the up-regulation of HCP5, which impacted the development of osteosarcoma (30). Moreover, Wu et al. (31) observed that overexpression of HCP5 could interact with miR-3619-5p, which upregulated peroxisome proliferative activated receptor gamma (PPARG) coactivator 1 alpha (PPARGC1A) through miR-3619-5p/the AMP-activated protein kinase (AMPK)/transcription complex peroxisome proliferator-activated receptor (PPAR) coactivator-1α (PGC1α)/enhancer-binding protein beta (CEBPB) axis, regulating 5-Fluorouracil (5-Fu) and oxaliplatin resistance in gastric cancer. This study surveyed 98 GC patients, 82 healthy donors, 21 gastritis patients, and 56 patients from diverse cancers. A diagnostic model consisting of serum HCP5 and existing indicators carcinoembryonic antigen (CEA) and carbohydrate antigen199 (CA199) was established to improve diagnostic efficacy. Besides, the role of HCP5 in the dynamic monitoring of tumors in GC patients has also been discovered.

## Methods

### Serum Samples Collection

From January 2016 to January 2021, Serum samples of 98 GC patients, 21 gastritis patients, 19 thyroid cancer (THCA) patients, 20 colorectal cancer (CRC) patients, 17 breast cancer (BRCA) patients, and 82 healthy donors were collected at the clinical laboratory of Affiliated Hospital of Nantong University (Nantong, China). All serum samples were stored in RNase-free test tubes at -80°C for later use. All samples mentioned above were collected under the ethics of the World Medical Association, and informed consent was obtained for experiments on human subjects. Besides, this study was approved by the Human Research Ethics Committee of Nantong University Hospital. (Ethics review report number: 2018-L055).

### RNA Extraction

According to the serum extraction kit (BioTeke, Wuxi, China), total RNA was extracted from 300µl serum. Then, 900µl lysis buffer was added, mixed by pipetting, vortexed for 1min, and incubated at 15-30°C for 5min. Adding 180μl chloroform in them and let them stand. After then, the serum samples were centrifuged at 12000rpm for 10min in a 4°C low-temperature high-speed centrifuge, followed by colorless water phases and organic phases. The colorless aqueous phase was then absorbed and transferred into a 1.5ml RNase-free tube, added to 700μl 70% ethanol, mixed upside down, and moved to the adsorption column. Centrifuged at 12000rpm for 1min and discarded the liquid. They were then adding 500μl deproteinized liquid, centrifuged at 12000rpm for 1min. After cleaning with 700μl rinsing solution, leaving for 2min and the liquid was transferred to a new 1.5ml RNase-free tube. Then, added 30μl RNase-free water in a warm bath, standing at room temperature for 2min, centrifuged at 12000rpm, and twice in 1min. After centrifugation, put it in the refrigerator at -80°C for later use.

### Complementary DNA (cDNA) Synthesis and Real-Time Fluorescence Quantitative Polymerase Chain Reaction (qRT-PCR)

The reverse transcription kit (Thermo Fisher Scientific) was used, adding 4µl 5×Reaction Buffer, 2µl 10mM deoxyribonucleotide triphosphate (dNTPs), 1µl Oligo (dT) Primer, 1µl RNase inhibitor (20U/µl), and 1µl Reverse Transcriptase (200U/µl) to form the reaction system, which was reverse-transcribed into cDNA with the total 11µl RNA solution. Then, we incubated the 20µl total system at 25°C for 5 minutes, 42°C for 60 minutes, 70°C for 5 minutes and stored the synthesized cDNA at -80°C when not in use. After that, qRT-PCR assays were performed on the Roche Lightcycler 480 (Roche, Switzerland), using SYBR Green I mix (Roche) as the fluorescent dyes for a total of 20µl system. The primers used in this article were all synthesized by RiboBio Corporation (Suzhou, China). The primer sequences are as follows: HCP5, 5’-TGAGAGCAGGACAGGAAAA-3’ (forward) and 5’-CCAACCAGACCCTAAGTGA-3’ (reverse); 18S ribosomal RNA (18S): 5’-CGCTCGCTCC TCTCCTACTT-3’ (forward) and 5’-CGGGTTGGTTTTGATCTGATAA-3’ (reverse).

The PCR cycling program included that activating the enzyme at 95°C for 10min, denaturation at 95°C for 15s, annealing at 60°C for 30s, then collecting fluorescence information at 80°C for a total of 45 cycles. The 2^-ΔΔCt^ method was used to calculate the relative expression of the target gene. ΔΔCt means the difference between the experimental group and the control group.

### Cell Culture

Human gastric epithelial cell (GES-1) and gastric cancer cell line (MKN-45, MGC-803, BGC-823, HGC-27) were purchased from the Cell Bank of the Chinese Academy of Sciences (Shanghai, China). Cells were cultured in RPMI-1640 (Corning, USA) containing 10% fetal bovine serum (FBS) (Gibco, USA) and 1% penicillin-streptomycin in a humid condition of 5% CO_2_ at 37°C.

### Cell Secretion Assay

The 1, 3, 5, and 7 days cell supernatants of MKN-45, MGC-803, and GES-1 were extracted. Furthermore, the expression level of lncRNA was later detected.

### Linear Verification Experiment

To verify the linearity of lncRNA, a healthy donor’s serum cDNA was diluted by 10, 10^2^, and 10^3^ times, respectively.

### Stability Verification Experiment

The mixed serum samples were placed at room temperature for 0, 6, 12, 18, and 24 h. Besides, repeated freezing and thawing for 0, 1, 3, 5, and 10 times to verify the stability of lncRNA.

### Agarose Gel Electrophoresis (AGE)

50×TAE 1:49 diluted to 1×, configured 2% gels, and waited for use. 1µl the loading buffer and 5µl products were added, then electrophoresed at 110-120V for about 30min. The accuracy of the product was verified by sequencing analysis.

### Cell Passage

Firstly, the original culture medium was discarded and washed 1-2 times with phosphate buffer saline (PBS). Secondly, cells in the T25 culture flasks were digested for 1min by adding about 1ml 0.25% trypsin-EDTA (Gibco, USA). Next, adding 2-3ml of the complete medium to culture flasks to stop digestion, rinsed, and suspended until all the cells were flushed down. They were then transferred to 15ml centrifuge tubes and centrifuged at 1000 rpm for 5min. Adding about 3ml complete medium to infiltrate the bottom of T25 culture flasks. While centrifugation was finished, we discarded the supernatant, adding about 4ml complete medium to suspend and evenly 2ml suspension to each bottle, mixed well, and then incubated in the condition with 5% CO_2_ at 37°C.

### Cell Cryopreservation

The original culture medium was discarded, rinsed 1-2 times with PBS, added approximately 1ml 0.25% trypsin-EDTA to digest cells in the T25 culture bottle for 1min. 2-3ml of complete medium was added to terminate digestion, transferred to a 15ml centrifuge tube and centrifuged for 5min at 1000rpm. Then, the suspension was transferred to the freezer tube and quickly placed in a refrigerator at -80°C for later use.

### Fluorescence In Situ Hybridization (FISH)

According to instructions of the manufacturer, the fluorescence *in situ* hybridization kit (RiboBio, Guangzhou, China) was used to repair and penetrate cells. 200μl prehybridization solution was incubated at 37°C for 30min, followed by 20μmol FISH probes/hybridization buffer for overnight incubation at 37°C. Rinsed each well with washing solution I, II, III and PBS in turn. 4**’**,6-Diamidino-2**’**-phenylindole (DAPI) was then dyed in the dark for 10min, and photos were taken under a fluorescence microscope. The HCP5 Fish probe was designed and synthesized by RiboBio (RiboBio, Guangzhou, China).

### Data Analysis

All statistics were analyzed using SPSS 20.0 software (IBM SPSS Statistics, Chicago, Illinois, USA) and GraphPad Prism V.8.00 software (GraphPad Software, La Jolla, USA). Meanwhile, all data were expressed as the mean ± standard deviation (SD) of values obtained in three independent experiments. According to the actual situation, independent sample t-test, one-way analysis of variance (ANOVA), and χ2 test were used for statistical analysis. The receiver operating characteristic curve (ROC) and the area under the ROC curve (AUC) were used to evaluate the diagnostic value of HCP5. Besides, bivariate logistic regression was used to analyze the diagnostic value of HCP5, CEA, and CA199. The ROC curve and AUC were obtained through non-parametric analysis. Youden index (Youden index = sensitivity + specificity - 1) was used as the expression value of serum HCP5. The overall survival (OS) was estimated by using the Kaplan-Meier method. All values of p<0.05 were considered to reach a statistically significant difference.

## Results

### The Innovative Detection Method of Serum HCP5

**Figure 1:**
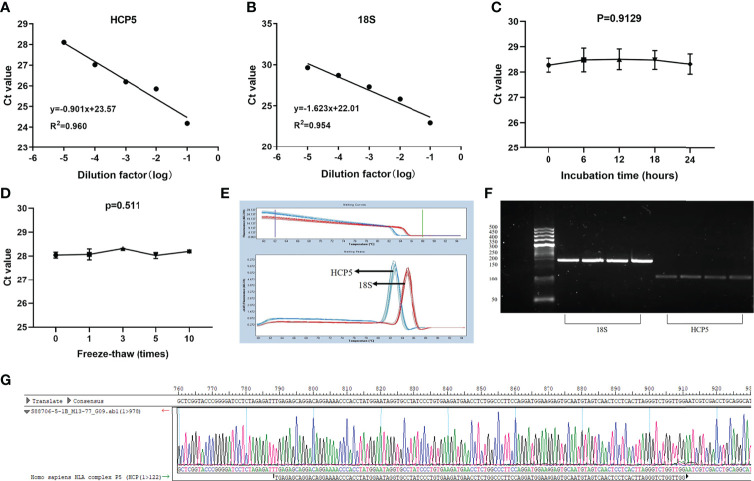
Methodology evaluation of HCP5 in GC serum samples. **(A, B)** Standard curves of serum HCP5 and 18S in a ten-fold serial dilution to show the linearity. **(C, D)** Stability of HCP5 under room temperature incubation time or multiple freeze-thaw cycles. Data were presented as raw Ct value (n=3). **(E)** The specificity of PCR products by the melting curve. **(F)** The validation of PCR products by the agarose gel electrophoresis. **(G)** The product sequence was verified by Sanger sequencing.

LncRNA, as a classic non-coding RNA, played a vital role in liquid biopsies as a biomarker. The literature confirmed that 18S had good linearity and stability compared with the reference genes glyceraldehyde 3-phosphate dehydrogenase, U6 small nuclear RNA (U6), β-actin (ACTB), and tubulin (TUB) in gastric cancer (32). Therefore, 18S was selected as the endogenous reference gene in this assay. To verify whether HCP5 was suitable for clinical laboratory analysis, we performed a methodological assessment of HCP5 detected by the qRT-PCR method. To verify its linearity, we built a continuous 10-fold dilution of cDNA and diluted the original serum cDNA concentrations 10, 10^2^ and 10^3^ times. Different concentrations of serum HCP5 were detected by qRT-PCR, and data analysis was performed (**Figure 1A**). At the same time, 18S was analyzed, as shown in **Figure 1B**. It could be seen that R^2^ of the standard curve of HCP5 was 0.960, and the regression equation was y=-0.901x+23.57, indicating that qRT-PCR was an effective method for detecting different concentrations of serum HCP5. In addition, we selected the mixture of serums for HCP5 precision determination. The results showed that the inter-and intra-assay coefficients of variation (CV) for HCP5 were 2.99% and 2.11%, respectively (**Table 1**). Secondly, to verify the stability of HCP5 in liquid biopsies, we placed the mixed standard human serum samples at room temperatures of 0, 6, 12, 18, and 24h. Similarly, we performed frozen-thawed cycles for another set of mixed samples 0, 1, 3, 5, and 10 times. All of these samples were stored in RNase-free tubes and kept at -80°C for later use. As shown in **Figures 1C**, **D**, when environmental conditions changed, the level of serum HCP5 remained significantly unchanged, indicating that HCP5 had good stability. Besides, the smooth unimodal melting curve also indicated the high specificity of amplified product serum HCP5 (**Figure 1E**). By analyzing the PCR products through AGE, the result showed that the band was 122bp and the accuracy was verified (**Figure 1F**). Sanger sequencing showed that the PCR product sequence was consistent with HCP5, with a sequence size of 122bp, further verifying the accuracy of the PCR method (**Figure 1G**). In this section, from all of these results, we found that the detection of HCP5 by qRT-PCR was susceptible and specific.

**Table 1:** Intra- and inter-assay reproducibility of HCP5 and 18S rRNA.

	HCP5	18s
**inter-assay CV,%**	**2.99%**	**3.28%**
**intra-assay CV,%**	**2.11%**	**2.58%**

### The Expression Level and Diagnostic Efficacy of Serum HCP5 in Gastric Cancer

**Figure 2:**
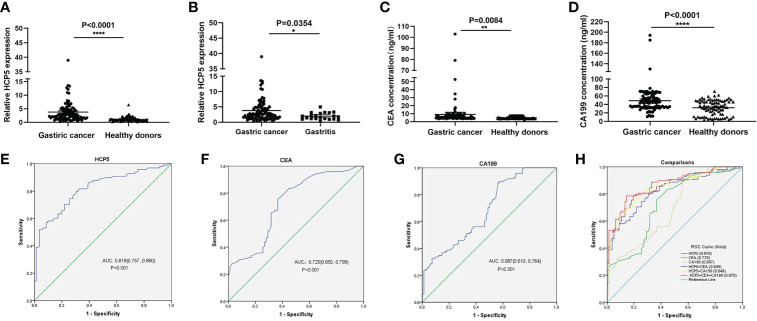
Concentrations of serum HCP5, CEA, and CA199 in GC cases and diagnostic efficacy**. (A)** Detection of serum HCP5 in GC patients (n=98), healthy donors (n=82). **(B)** Detection of serum HCP5 in GC (n=98) and gastritis (n=21) patients. **(C)** The concentrations of serum CEA. **(D)** The concentrations of serum CA199. ^*^P < 0.05, ^**^P < 0.01, ^****^P < 0.001, NS means no statistically difference. **(E)** The ROC curve of serum HCP5 in GC cases. **(F)** The ROC curve of serum CEA in GC cases. **(G)** The ROC curve of serum CA199 in GC cases. **(H)** The diagnostic efficacy of the combined diagnosis.

To verify whether HCP5 can be used as an emerging GC diagnostic marker. We detected the expression of serum HCP5 in 98 GC patients and 82 healthy donors by qRT-PCR. The results showed that the expression level of serum HCP5 in GC patients was significantly higher than that in healthy donors (**Figure 2A**). In addition, HCP5 can clearly distinguish patients with gastritis and gastric cancer (**Figure 2B**). Besides, CEA and CA199 are usually widely used for screening and auxiliary diagnosis of GC. As shown in **Figures 2C, D**, the expression levels of serum CEA and CA199 in GC patients were also higher than those in healthy controls. The ROC curves showed that the area under the curve of serum HCP5 detected by qRT-PCR was 0.818 (95% CI:0.757-0.880 P<0.001) (**Figure 2E**). Under this cut-off value, the sensitivity was 80%, and the specificity was 70% (**Table 2**). Moreover, the clinical indicators CEA and CA199 area under the curve were 0.725 (95% CI:0.650-0.799, P<0.001) and 0.687 (95% CI:0.610-0.764, P<0.001), respectively (**Figures 2F, G**). Besides, we also analyzed the expression level of HCP5 and the comprehensive diagnostic analysis between CEA and CA199. As mentioned above, compared with the separate detection, the three combined diagnoses provided the high AUC 0.870 (95% CI:0.819-0.921, P<0.001) in the distinction between gastric cancer and healthy donors (**Figure 2H**). Then, through logistic regression analysis, the diagnostic efficiency of several serum biomarkers was discussed. Besides, compared with single detection, combined detection can better distinguish GC patients from healthy donors, especially when compared with the combined diagnosis model of HCP5, CEA, and CA199, the combination of which can increase sensitivity and specificity, which could reach 81% and 79%, respectively (**Table 2**). The above data showed that the single diagnosis of HCP5 had a significant AUC, and the combined diagnosis of HCP5, CEA, and CA199 can improve the diagnosis efficiency of GC.

**Table 2:** Combination of serum HCP5, CEA and CA199 levels significantly improves the diagnostic sensitivity between GC patients and healthy donors.

	SEN,%	SPE,%	ACCU,%	PPV,%	NPV,%
HCP5	0.80(78/98)	0.70(57/82)	0.75(135/180)	0.76(78/103)	0.74(57/77)
CEA	0.67(66/98)	0.66(54/82)	0.67(120/180)	0.70(66/94)	0.63(54/86)
CA199	0.56(55/98)	0.59(48/82)	0.57(103/180)	0.62(55/89)	0.53(48/91)
HCP5+CEA	0.79(77/98)	0.80(66/82)	0.79(143/180)	0.83(77/93)	0.76(66/87)
HCP5+CEA+CA199	0.81(79/98)	0.79(65/82)	0.80(144/180)	0.81(79/98)	0.77(65/84)

### Origin of HCP5 in GC Samples

**Figure 3:**
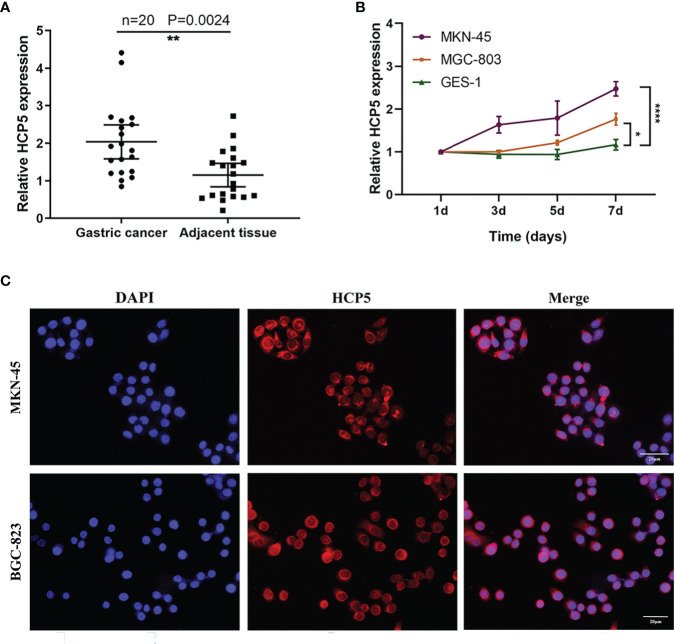
The origin of serum HCP5 in GC cases. **(A)** The expression level of HCP5 in 20 pairs of GC tissues. **(B)** HCP5 was secreted into the culture medium by MKN-45 and MGC-803 cells in a time-dependent manner. *P < 0.05, **P < 0.01, ****P < 0.001. **(C)** The FISH assay of HCP5 in MKN-45 and BGC-823 cells. Scale bars: 20μm.

To understand the overall expression level of HCP5 in GC, we collected 20 pairs of tissue samples. The result showed that HCP5 was significantly highly expressed in GC tissues (**Figure 3A**). Because of the high expression of HCP5 in serum GC, we assumed whether HCP5 was secreted by GC cells. Then, we detected the expression level of HCP5 in two GC cell lines (MKN-45, MGC-803) and normal control cells GES-1 for 1, 3, 5, and 7 days, respectively (**Figure 3B**). The results showed that compared with normal cell lines, HCP5 increased in MKN-45 and MGC-803 over time, especially in MKN-45. Hence, we speculated that the high expression of HCP5 in peripheral blood might be derived from the secretion of some tumor cells. Furthermore, we detected the subcellular localization of lncRNA HCP5, and the results showed that HCP5 was mainly distributed in the cytoplasm of MKN-45 and BGC-823 cells (**Figure 3C**).

### Diagnostic Efficacy of Serum HCP5 in Various Cancer Types and Distinguishing Between Gastritis and Healthy Donors

**Figure 4:**
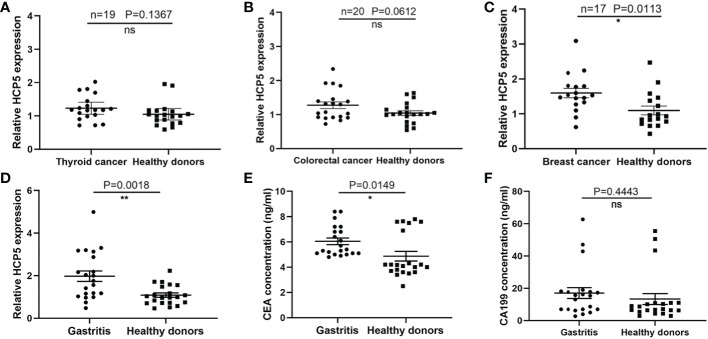
The specificity of serum HCP5 in GC and differentiating from gastritis and healthy donors. **(A)** Detection of serum HCP5 in 19 thyroid cancer patients. **(B)** Detection of serum HCP5 in 20 colorectal cancer patients. **(C)** Detection of serum HCP5 in 17 breast cancer patients. **(D)** The expression levels of HCP5 in gastritis patients (n=21) and healthy donors (n=21). **(E)** The concentrations of CEA in gastritis patients (n=21) and healthy donors (n=21). **(F)** The concentrations of CA199 in gastritis patients (n=21) and healthy donors (n=21). *P < 0.05, **P < 0.01, NS means no statistically difference.

To verify the specificity of HCP5 expression levels, serum expression levels of 19 thyroid cancer patients, 20 colorectal cancer patients, and 17 breast cancer patients were examined. The results showed that HCP5 expression was not statistically significant not only in thyroid cancer but also in colorectal cancer, and in breast cancer had a little significance (**Figures 4A–C**). At the same time, we detected expression levels of serum HCP5, CEA, and CA199 in 21 patients with gastritis patients and 21 healthy donors, respectively (**Figures 4D–F**). Collectively, the results indicated that serum HCP5 could distinguish gastritis patients from healthy donors. Thus, it can be seen HCP5 can be regarded as a specific biomarker for GC diagnosis.

### Correlation Between Serum HCP5 Expression and GC Clinicopathological Parameters

The clinicopathological parameters of 98 patients with GC were summarized in **Table 3**. Based on the cut-off value of serum HCP5, 98 GC patients were divided into the high-value and low-value groups. Chi-square test showed that although we did not find any correlation with serum HCP5 expression in pathological parameters such as gender, age, tumor size, TNM stage, distant metastasis, and CEA. Nevertheless, the high expression of serum HCP5 was correlated with differentiation (P<0.05), lymph node metastasis (P<0.05), and nerve invasion (P<0.05), indicating that serum HCP5 expression was correlated with some GC clinicopathological parameters.

**Table 3:** The Correlation between HCP5 expression and clinicopathologic parameters of GC patients.

Characteristics	Total	HCP5		P value
		High expression n = 49	Low expression n = 49	
Gender				0.306
Male	57	31	26	
Female	41	18	23	
Age (years)				0.541
≥60	43	20	23	
<60	55	29	26	
Differentiation				0.026*
Poorly/moderately poorly	53	32	21	
Well/medium-well	45	17	28	
Tumor size (cm)				0.258
≥5	27	11	16	
<5	71	38	33	
TNM stage				0.840
TI-TII	51	26	25	
TIII-TIV	47	23	24	
Lymph node metastasis				0.043*
Positive	50	30	20	
Negative	48	19	29	
Distant metastasis				0.106
Positive	50	29	21	
Negative	48	20	28	
Nerve invasion				0.026*
Positive	51	31	20	
Negative	47	18	29	
CEA (ng/ml)				0.389
≥5.0	66	35	31	
<5.0	32	14	18	

Statistical analyses were carried out using Pearson χ^2^ test. *P < 0.05 was considered significant.

### Tumor Dynamic Monitoring of Serum HCP5 in GC Patients

**Figure 5:**
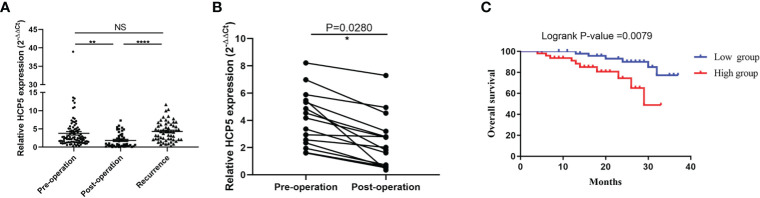
Serum HCP5 in monitoring tumor dynamics in GC patients. **(A)** Detection of serum HCP5 expression in GC pre-operation patients (n=98), post-operation patients (n=46) and recurrence patients (n=57). **(B)** Altered expression of serum HCP5 in 15 paired samples preoperatively and postoperatively. **(C)** The survival curve of patients with GC. ^*^P < 0.05, ^**^P < 0.01, ^****^P < 0.001, NS means no statistically difference.

Due to previous reports and our current research, circulating lncRNAs may be secreted by tumor cells, and they would return to an average level after surgery. To verify whether the expression of serum HCP5 was related to tumor dynamic monitoring, we compared serum HCP5 in unpaired samples, including 98 patients with primary gastric cancer, 46 patients with surgical treatment, and 57 patients with tumor recurrence. It was found that the serum HCP5 expression of patients after treatment was significantly lower than that of GC patients and relapsed patients before treatment (**Figure 5A**). Meanwhile, we investigated the differences in serum HCP5 levels in 15 pairs of GC patients before and after surgery. The results showed the expression level of HCP5 after gastrectomy was significantly lower than that of patients with primary GC, which showed that HCP5 could be used for dynamic monitoring (**Figure 5B**). Besides, the survival curve revealed that the overall survival rate of patients with high HCP5 expression was significantly lower than that of patients with low HCP5 expression (P=0.0079) (**Figure 5C**). To sum up, we believed that serum HCP5 could be regarded as a new marker for early diagnosis of GC and tumor dynamic monitoring.

## Discussion

Gastric cancer is the common cancer with high mortality and morbidity. Recently, the treatment effect and prediction were still poor and low. Surgical resection was still the primary treatment at present. lncRNA can be divided into sense, antisense, intronic, bidirectional transcripts, intergenic and enhancer RNAs (33). Current studies have shown that lncRNA carried out strict regulation and abnormal expression during the development of different cancers, which can be divided into (1) chromatin regulation; (2) regulation of histone modification; (3) regulation of DNA methylation; (4) interaction with transcription factors; (5) regulation of genomic tissue through enhancer cycle; (6) post-transcriptional regulation (34). For example, kinds of literature have shown that NEAT1 affects chromatin remodeling, increased histone acetylation level, promoted aldehyde dehydrogenase 1 family (ALDH1) and c-Myc expression, and improved drug resistance and tumor dryness 5-FU (35). Zhang et al. (36) found that lnc-LALC bonded to enhancer of zeste homolog 2 (EZH2), recruited DNA methyltransferase (DNMT) to Leucine zipper putative tumor suppressor 1 (LZTS1) promoter, and changed the expression of LZTS1. Another literature reported that in ischemic heart disease, the combination of lncCIRBIL and BCL2-related transcription factor1 (Bclaf1) had a protective effect on I/R damage and was a potential target for treating ischemic heart disease (37). What’s more, it also has been convinced that the chromatin cyclization between enhancers E1 and E2 and lncRNA promoter played a co-regulatory role in prostate cancer (38). Furthermore, Wang et al. (39) found that linc00336 can act as an endogenous sponge of miRNA to inhibit lung cancer hypertrophy. Simultaneously, more and more pieces of literature have confirmed that lncRNA can be used as a potential biomarker for tumor screening and prognosis monitoring (20–24). Inspired by this idea, this study was devoted to finding suitable tumor biomarkers.

HCP5, also named P5-1, is located on chromosome 6p21.33. Previous experiments have shown that HCP5 can participate in regulating various tumors, such as HCP5, which was mediated by transforming growth factor β (TGF β) and regulated by recombinant SMAD family member 3 (SMAD3) transcription, could promote the growth and metastasis of lung adenocarcinoma (LUAD) tumors (40). Moreover, HCP5 can encourage the development of cervical cancer by inhibiting microRNA-15a regulating metastasis-associated in colon cancer-1 (MACC1) (30). The above studies illustrated that HCP5 was a regulator in the process of tumor development, and it may become a predictor of tumor diagnosis and treatment. At present, real-time fluorescence quantitative PCR (41), microarray (42), and next-generation sequencing (NGS) (43) are often used for quantitative analysis of ncRNA. qRT-PCR was considered a reliable method for detecting non-coding RNA gene expression because of its high sensitivity, speed, and efficiency, as the small sample requirements and ease of interpretation of the results were significant advantages (44). Consequently, in this study, we chose the qRT-PCR method to evaluate serum HCP5 expression, and 18S was selected as the internal reference. The results showed that the expression of HCP5 in GC patients by this method had good linearity, stability, specificity, and repeatability. Besides, large serum samples of gastric cancer detected by qRT-PCR showed that HCP5 could not only significantly distinguish between GC patients and healthy donors, but also could distinguish GC patients from gastritis patients well. The diagnostic efficiency of HCP5 alone was substantially higher than that of CEA and CA199, and the combined diagnosis of HCP5, CEA, and CA199 can improve the diagnostic efficiency of gastric cancer. Then, we detected the expression level of serum HCP5 in thyroid, colorectal, and breast cancer, found that its expression in the serum GC was the most specific. At the same time, we also detected the expression level of serum HCP5 in gastritis and normal controls. Compared with the typical clinical diagnostic indicators CEA and CA199, the results showed that serum HCP5 could better distinguish gastritis patients from healthy donors. From the above experiments, it can be seen that HCP5 showed a remarkable advantage as a specific biomarker for the diagnosis of GC. Besides, clinicopathological parameters also showed that the high expression of serum HCP5 in GC was notably correlated with differentiation (P<0.05), lymph node metastasis (P<0.05), and nerve invasion (P<0.05), indicating that serum HCP5 expression was correlated with some GC clinicopathological parameters. Moreover, the statistics of 20 pairs of GC tissues showed that HCP5 had a conspicuous trend of high expression. What’s more, previous literature reported a pronounced correlation between the expression of circulating biomarkers in blood and cells (45). To verify whether HCP5 was secreted by tumor cells, we also examined the expression of HCP5 in GES-1, MGC-803, and MKN-45. We found that the expression of HCP5 in MGC-803 and MKN-45 was dramatically increased, especially in MKN-45. It was speculated that HCP5 might come from the secretion of tumor cells and was mainly related to tumor metastasis. Meanwhile, the FISH assay showed that HCP5 was mainly present in the cytoplasm of MKN-45 and BGC-823. Besides, by comparing the expression of serum HCP5 in patients with primary gastric cancer, patients undergoing surgery, and patients with postoperative recurrence, we found that serum HCP5 rebounded in patients with tumor recurrence and it had the ability to monitor tumor dynamics. Meanwhile, we also detected the expression level of HCP5 in 15 pairs of GC patients before and after an operation. The results showed that the expression level of HCP5 after gastrectomy was strikingly lower than that in patients with primary GC, which indicated that HCP5 could be a specific biomarker and played an important role in dynamic monitoring. The survival curve showed that the OS rate of patients with high serum HCP5 was significantly lower than that of patients with low expression of serum HCP5. It showed a positive correlation between a high level of HCP5 and poor survival and prognosis of GC.

In short, our study showed that the increase of serum HCP5 could significantly distinguish between patients with primary gastric cancer and healthy controls, and the combined diagnosis of HCP5, CEA, and CA199 had high diagnostic efficiency. At present, gastritis is mainly divided into gastroscopy and laboratory examination, which included gastric juice analysis, pepsinogen test, serum gastrin test, and Helicobacter pylori test. There may be no significant difference in laboratory indexes among different types and locations of Lauren tumors, which had some limitations (46). For Helicobacter pylori testing, studies have shown that bacteria could be eliminated in atrophic gastritis development, so it also had certain limitations (47). Hence, it was indispensable to find other specific serum biomarkers combined with other preventive measures. Compared with the traditional clinical indexes, serum HCP5 can better distinguish gastritis from healthy donors. Thus, it can be concluded that serum HCP5 may be a potential biomarker in dynamic monitoring of gastric cancer and tumor. Nevertheless, there were some limitations in this study. The samples of this study were only limited to one hospital in China. If conditions permit, serum samples from patients in multiple hospitals could be collected for further study. In addition, due to the limited sample size of patients with gastritis, it was necessary to increase further gastritis samples and long-term observation of patients with gastritis to obtain new insights into the potential mechanism of the diagnostic and prognostic value of HCP5.

## Data Availability Statement

The original contributions presented in the study are included in the article/**Supplementary Material**. Further inquiries can be directed to the corresponding author.

## Ethics Statement

The studies involving human participants were reviewed and approved by the ethics committee of the Affiliated Hospital of Nantong University (ethical review report number: 2018-L055). The patients/participants provided their written informed consent to participate in this study. Written informed consent was obtained from the individual(s) for the publication of any potentially identifiable images or data included in this article.

## Author Contributions

SQ designed the experiment, conducted the data analysis, made charts, and wrote the article. LY performed the experiment and conducted the data collection. SK gave some suggestions and carried out the revision of the article. YX gave advice for some charts and participated in the final revision. BL and SJ provided resources and guidance for the paper. All authors contributed to the article and approved the submitted version.

## Funding

This project was supported by grants from the National Natural Science Foundation of China [grant number: 81871720, 82072363], and Jiangsu Province “science, education, and health” key discipline [grant number: ZDXKB2016011].

## Conflict of Interest

The authors declare that the research was conducted in the absence of any commercial or financial relationships that could be construed as a potential conflict of interest.

## Supplementary Material

Supplementary Figure 1
**(A)** The ROC curve of the poorly, moderately poorly groups and well, medium-well groups. **(B)** The ROC curve of the lymph node metastasis positive and negative groups. **(C)** The ROC curve of the nerve invasion positive and negative groups.

Supplementary Figure 2
**(A)** Serum HCP5 expression of differentiation. **(B)** Serum HCP5 expression of lymph node metastasis. **(C)** Serum HCP5 expression of nerve invasion.

Supplementary Figure 3 The time-dependent studies on HGC-27, BGC-823, and GES-1 cells.

Supplementary Figure 4
**(A)** The ROC curve of the TIII-TIV group. **(B)** The ROC curve of the TI-TII group.

## Glossary

GC: gastric cancer
lncRNA: long non-coding RNA
mRNA: messenger RNA
miRNA: microRNA
ceRNA: competitive endogenous RNA
CAD: coronary artery disease
CRC: colorectal cancer
HCP5: Histocompatibility leukocyte antigen complex P
MICA: MHC class I polypeptide related sequence A
MICB: MHC class I polypeptide related sequence B
MHCI: MHC class I chain-related gene
SP1: the transcription factor 1
PPARG: peroxisome proliferative activated receptor gamma
PPARGC1A: PPARG coactivator 1 alpha
AMPK: the AMP-activated protein kinase
PPAR: peroxisome proliferator-activated receptor
PGC1α: PPAR coactivator-1α
CEBPB: enhancer-binding protein β
5-Fu: 5-Fluorouracil
CEA: carcinoembryonic antigen
CA199: carbohydrate antigen199
THCA: thyroid cancer
BRCA: breast cancer
cDNA: Complementary DNA
qRT-PCR: real-time fluorescence quantitative polymerase chain reaction
dNTPs: deoxyribonucleotide triphosphate
18S: 18S ribosomal RNA
FBS: fetal bovine serum
AGE: Agarose Gel Electrophoresis
PBS: phosphate buffer saline
FISH: fluorescence *in situ* hybridization
DAPI: 4’&rsquo;,6-Diamidino-2’-phenylindole
SD: standard deviation
ANOVA: one-way analysis of variance
ROC: receiver operating characteristic curve
AUC: area under the ROC curve
OS: overall survival
U6: U6 small nuclear RNA
ACTB: β-actin
TUB: tubulin
CV: coefficients of variation
STAD: stomach adenocarcinoma
ALDH1: aldehyde dehydrogenase 1 family
EZH2: enhancer of zeste homolog 2
DNMT: DNA methyltransferase
LZTS1: Leucine zipper putative tumor suppressor 1
Bclaf1: BCL2-related transcription factor1
TGF β: transforming growth factor β
SMAD3: recombinant SMAD family member 3
LUAD: Lung adenocarcinoma
MACC1: metastasis-associated in colon cancer-1
NGS: next-generation sequencing
