# Supplementary figures and images for: LncRNA HCP5 : A Potential Biomarker for Diagnosing Gastric Cancer
Source: Front Oncol. 2021 Jun 18;11:684531. doi: 10.3389/fonc.2021.684531 (PMC8252797; doi:10.3389/fonc.2021.684531)

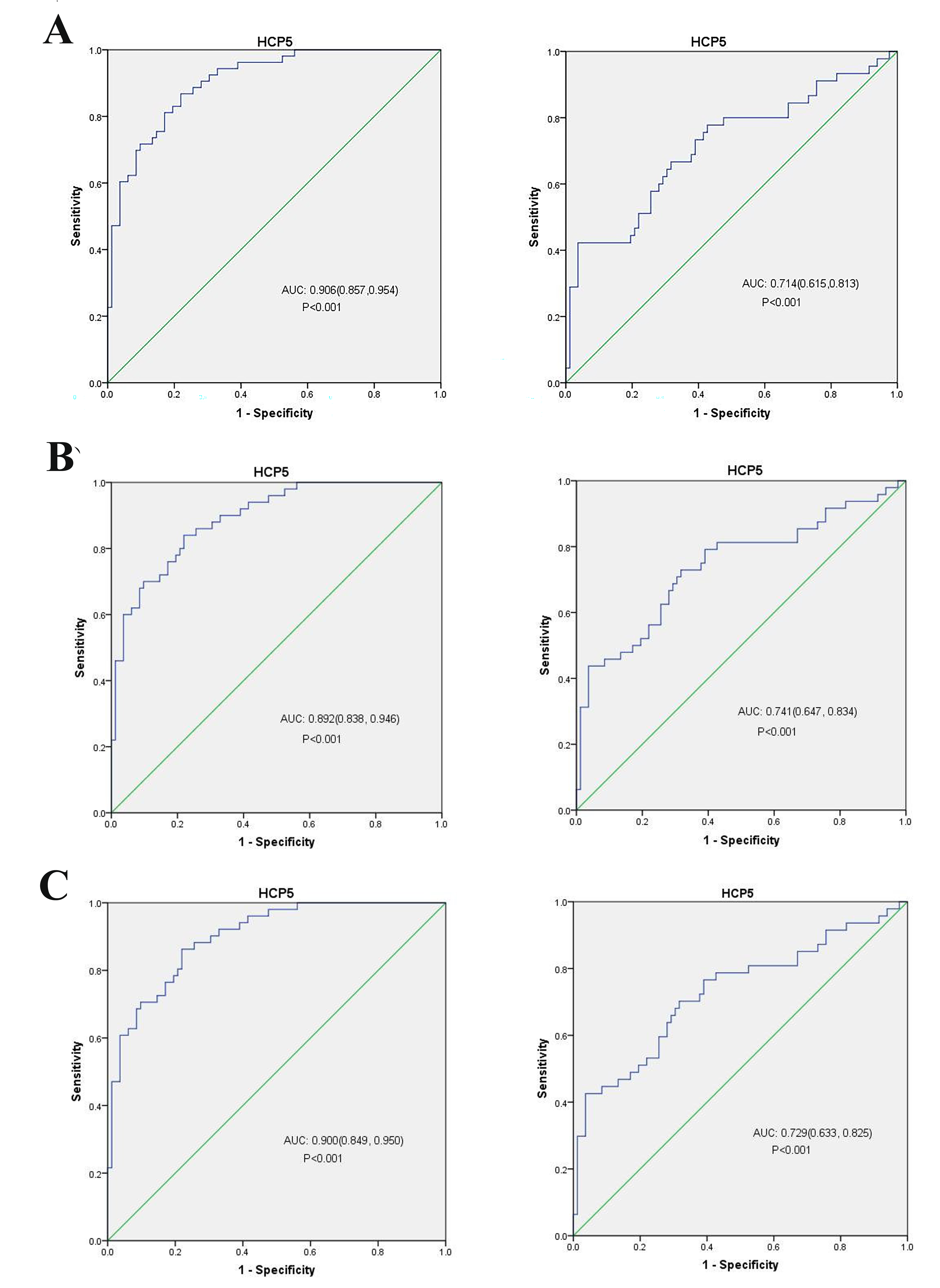

Supplement: Supplementary Figure 1 — (A) The ROC curve of the poorly, moderately poorly groups and well, medium-well groups. (B) The ROC curve of the lymph node metastasis positive and negative groups. (C) The ROC curve of the nerve invasion positive and negative groups. [file Image_1.tif]

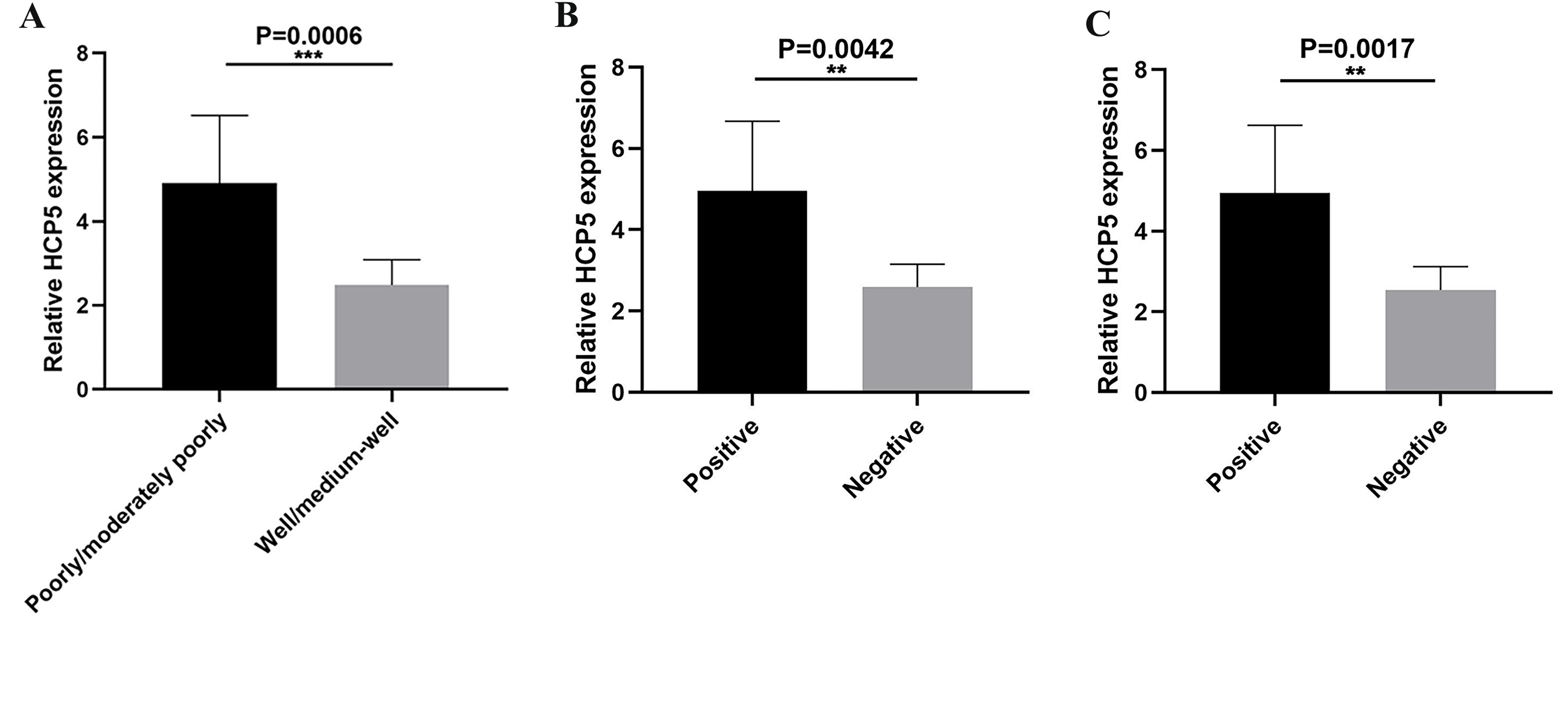

Supplement: Supplementary Figure 2 — (A) Serum HCP5 expression of differentiation. (B) Serum HCP5 expression of lymph node metastasis. (C) Serum HCP5 expression of nerve invasion. [file Image_2.tif]

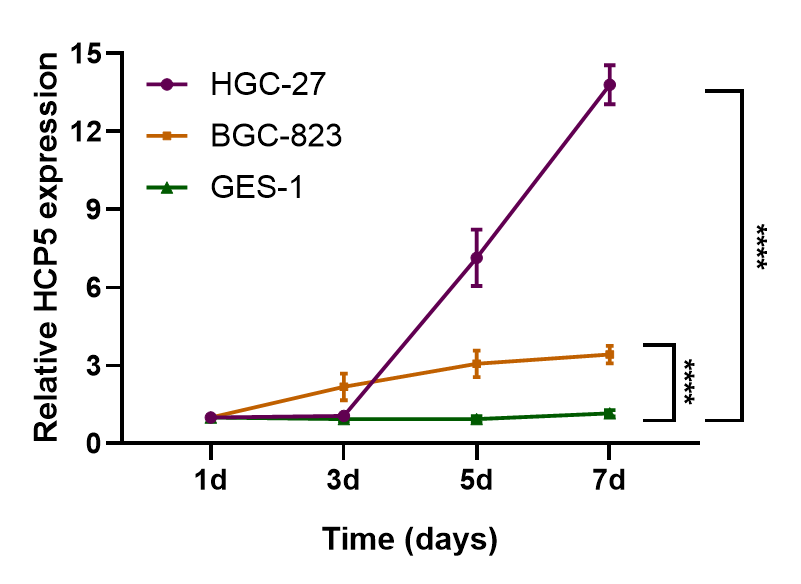

Supplement: Supplementary Figure 3 — The time-dependent studies on HGC-27, BGC-823, and GES-1 cells. [file Image_3.png]

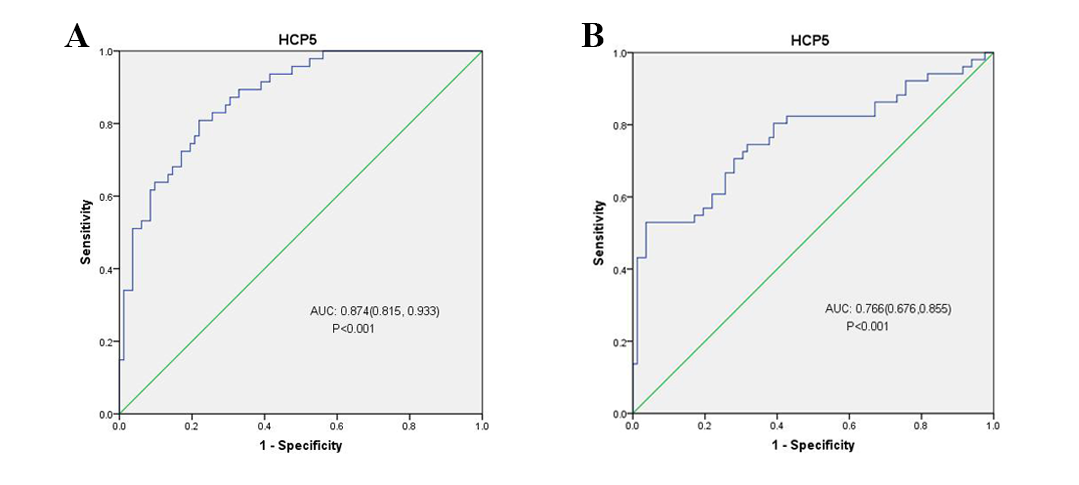

Supplement: Supplementary Figure 4 — (A) The ROC curve of the TIII-TIV group. (B) The ROC curve of the TI-TII group. [file Image_4.tif]
